# The Importance of Nodule Size in the Management of Ruptured Thyroid Nodule After Radiofrequency Ablation: A Retrospective Study and Literature Review

**DOI:** 10.3389/fendo.2021.776919

**Published:** 2021-11-26

**Authors:** Wen-Chieh Chen, Sheng-Dean Luo, Wei-Chih Chen, Chen-Kai Chou, Yen-Hsiang Chang, Kai-Lun Cheng, Wei-Che Lin

**Affiliations:** ^1^ Division of Endocrinology and Metabolism, Department of Internal Medicine, Kaohsiung Chang Gung Memorial Hospital, and Chang Gung University College of Medicine, Kaohsiung, Taiwan; ^2^ Department of Otolaryngology, Kaohsiung Chang Gung Memorial Hospital, and Chang Gung University College of Medicine, Kaohsiung, Taiwan; ^3^ Department of Nuclear Medicine, Kaohsiung Chang Gung Memorial Hospital, and Chang Gung University College of Medicine, Kaohsiung, Taiwan; ^4^ Department of Medical Imaging, Chung Shan Medical University Hospital, Taichung, Taiwan; ^5^ School of Medical Imaging and Radiological Sciences, Chung Shan Medical University, Taichung, Taiwan; ^6^ Department of Diagnostic Radiology, Kaohsiung Chang Gung Memorial Hospital, and Chang Gung University College of Medicine, Kaohsiung, Taiwan

**Keywords:** thyroid nodule, ultrasound, radiofrequency ablation, complication, nodule rupture

## Abstract

**Background:**

Nodule rupture is a relatively uncommon yet severe complication of radiofrequency ablation (RFA). When nodule rupture occurs, determining suitable therapeutic management is a critical issue. A study herein aimed to identify the predictive factors affecting the management of post-RFA nodule rupture.

**Methods:**

Post-RFA nodule rupture data of 9 patients were enrolled from 2 medical centers. A literature investigation was performed, uncovering nodule rupture data of 17 patients. A total of 26 patients were analyzed and divided into two groups, categorized as patients requiring either invasive or conservative therapeutic management. Data including initial symptoms, imaging, therapeutic management, and prognosis were reviewed and compared between the two groups.

**Results:**

Significant differences in nodule diameter, and the ablation time of the course prior to rupture (RUP time) were noted between the two groups (p = 0.045 and 0.008, respectively). Logistic regression analysis indicated the initial nodule diameter and RUP time significantly affected the requirement of invasive treatment (OR 1.99 and 1.11, respectively). Considering practicality, when a nodule with an initial maximum diameter of >4.5cm ruptured, invasive management was suggested (sensitivity 69% and specificity 79%).

**Conclusion:**

Though nodule ruptures can be managed conservatively, a ruptured nodule with an initial maximum diameter of >4.5cm may require invasive management. Understanding the significant clinical and imaging features will help physicians make an appropriate risk assessment to determine the correct treatment in a timely manner.

## Introduction

Radiofrequency ablation (RFA) is an effective procedure with a low complication rate (1–3), indicating for those patients who with benign thyroid nodule (4, 5). However, risks exist with every procedure, thus planned follow-ups are essential. RFA-associated complications include voice changes, skin burns, hematoma, brachial plexus injury, nodule rupture, and transient hyper-/hypo-thyroidism (5, 6). The complication rate for benign nodules was 3.3%-5.6% (1, 6–8). More extensive physician experience is directly related to fewer complications (9).

Among complications, nodule rupture sometimes requires invasive interventions, including aspiration, drainage or even debridement or lobectomy (6, 10, 11). In one recently-published study involving 12 patients with thyroid nodule rupture after RFA, the nodule rupture could be further classified by the location of rupture (12). Although the majority of ruptures can be managed conservatively, some indeed require further intervention, creating a burden for both patients and surgeons. Currently, studies of the clinical factors affecting treatment post rupture are limited. The study aimed to evaluate the relationship among management, the clinical manifestations, and imaging features. The data of 9 patients with post-RFA goiter rupture were collected from two of the largest thyroid RFA centers in our country, while the 17 other ones were collected from literature review. The total 26 patients were divided into two groups, based on having received invasive or conservative treatment. The parameters affecting initial management were determined, and the odds ratio of each parameter was calculated by logistic regression analysis.

## Materials and Methods

### Literature Review

A bibliographic search was performed on PubMed, Embase (updated to November 2020) using the keywords “benign thyroid nodule”, “complication” and “radiofrequency ablation”. Articles were included if (1) consisted of patients experiencing nodule rupture after RFA for benign thyroid nodule; (2) the management section mentioned specific treatment (including antibiotics, debridement, and surgery) and treatment response; (3) written in English. The following information was extracted from each study: author, publication year, country, participant characteristics (complication rate of ruptures, the volume and the largest diameter of ruptured nodule, mean days to rupture and management).

### Patient Evaluations

Data of 10 adult patients with nodule rupture after RFA at two medical centers between 2017 and 2019 (total 818 RFA sessions) were evaluated. Data of 9 confirmed eligible and included in the study (1 lost follow-up). The 9 patients all completed the 1-year follow-up, and every nodule was surveyed by ultrasonography (US). All nodules were confirmed benign by at least two fine needle aspirations or core needle biopsy prior to RFA (4, 13). Data including baseline characteristics, initial symptoms of rupture, imaging, management, and prognoses were analyzed. The ablation time of the course prior to rupture was defined as the RUP time. The volumes of tumors were calculated (V = πabc/6; V: volume; a/b/c: transverse/vertical/antero-posterior diameter). The composition of nodule was classified as either predominantly solid (solid component >50%) or cystic (solid component between 10-50%). This retrospective study was approved by the Institutional Review Boards of the two medical centers. Finally, to explore the potential predictive factors of need for invasive management, the available 17 patient data from previous studies (10, 12) (from the related facilities where the two radiologists received RFA training before) and the 9 patient’s data in our institutions were integrated for analysis.

### RFA Procedures

Real-time US-guided RFAs were performed in an outpatient setting by two radiologists. The two experienced radiologists had similar training process, and each performed over 100 sessions of RFA per year. Since RFA is preferable for solid-predominant nodules (7, 14, 15), the cystic content was aspirated prior to RFA if feasible (16, 17). The critical triangle, consisting of the carotid space, trachea, and esophagus was carefully monitored. An internally cooled electrode (18 gauge, with 5 mm, 7 mm or 1-cm active tip) with RF generator (VIVA, STARmed and M2004, RF Medical) were used. 2% lidocaine was injected slowly to the thyroid capsule for local anesthesia. The RF needle was inserted in the lesion using the trans-isthmic approach and moving shot technique (4, 18). Ablation was terminated when the whole nodule had been changed to transient hyperechoic zones. Mild compression at the treatment site was performed for 10-20 minutes. Follow-ups for US at 1 week, 1 month, 3 months, 6 months, 9 months, and 1 year after RFA were scheduled to observe nodule characteristics and volume reduction.

### Statistical Analysis

The presentation of data included both categorical and continuous variables. Statistical analyses were performed using SPSS Version 23 software (SPSS, Inc.). The 26 patients were divided into two groups according to whether or not invasive treatment was needed after rupture. The chi-square test for evaluation of the categorical variables, and independent- sample t- test for the continuous variables with normal distribution were performed. The Mann-Whitney U test was used for continuous variables with non-normal distribution. After finding statistically significant parameters, two-tailed Pearson’s correlation was used to evaluate the correlation between parameters. Logistic regression analysis was used to calculate the odds ratio (OR) and 95% confidence interval (CI) of the above parameters with statistical significance. P <0.05 was considered significant. The receiver operating characteristic (ROC) curve analysis with Youden’s index was performed to determine the cut-off point of these parameters regards to need for invasive management.

## Results

**Figure 1:**
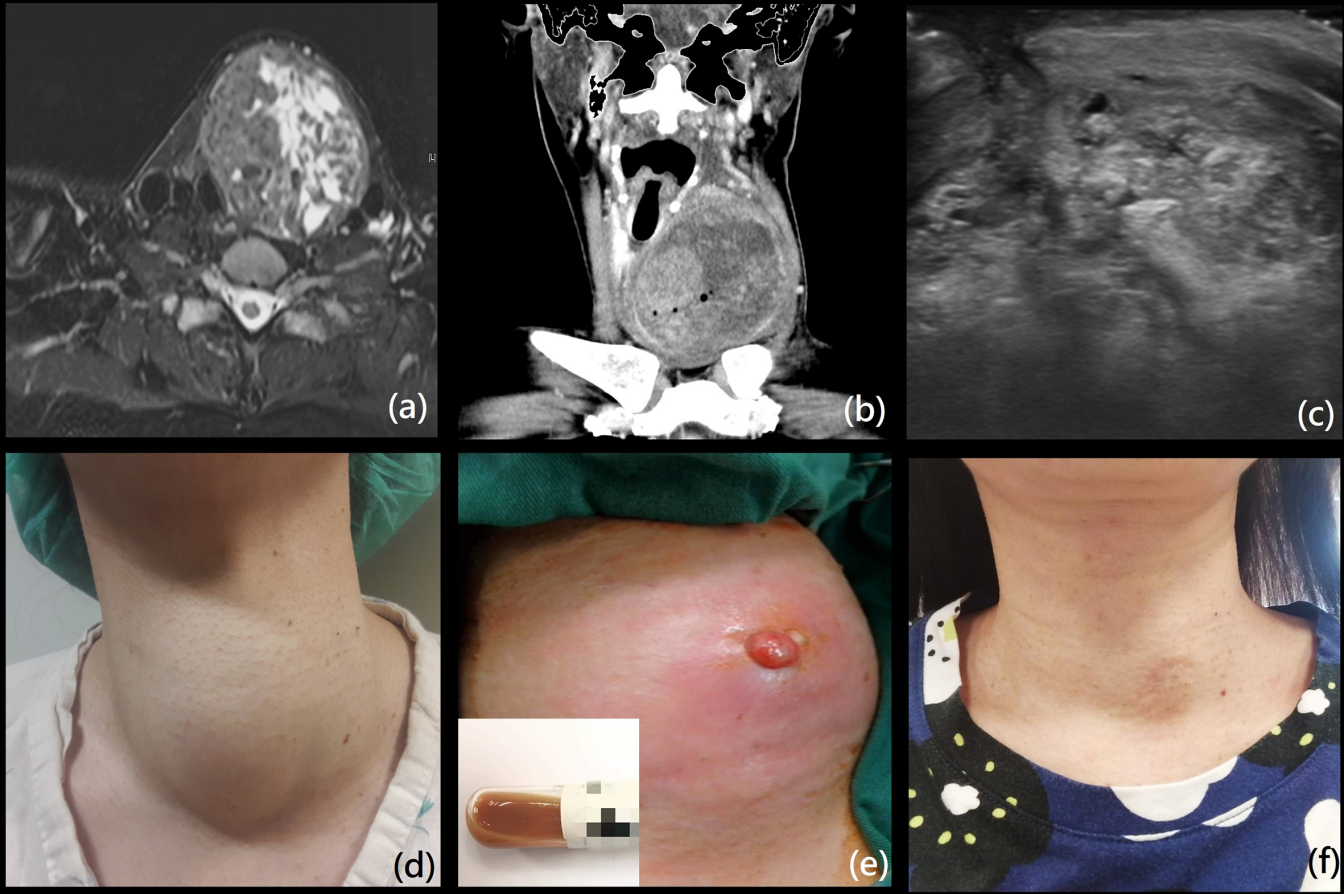
**(A)** A 44-year-old woman presented with a huge mixed-intensity thyroid mass, with initial size of 8.1 x 7.9 x 5.9 cm (volume 198ml) on MRI. She received one RFA session with total energy 25.14 kCal and procedure time of 50 min and 43 sec. **(B)** Sudden swelling and erythema on the left neck was noted 19 days after the RFA. Coronal view of computed tomography demonstrated necrosis of nodule with anterior capsule disruption with fluid extension subcutaneously (anterior type). The patient was admitted for intravenous antibiotics and surgical debridement; brown mucous content without hemorrhage were noted. She was hospitalized for 7 days. **(C)** The lesion gradually regressed to size of 4.5 x 2.9 x 3.7 cm at 1 month after surgery on echo. **(D–F)** The patient’s appearances on the time prior to RFA, at debridement, and two years after RFA. We have obtained the patient’s consent to apply her photographs and information in the article.

The baseline characteristics of the 9 cases in the study (7 females and 2 males, with mean age 38.9 ± 7.3 years) are summarized in **Table 1**. The complication rate of nodule rupture was 1.1% (9/818). The ruptured nodules were predominantly solid (mean volume 73.9 ml; maximum 198ml). The mean ablation time and mean total energy per RFA session were 29 minutes (range, 9.4 to 50.7) and 16.6 kcal (range, 1.5 to 33.3), respectively. The median energy per RFA session was 50 W. The volume reduction rate (VRR) was 83.6 ± 5.8%. All patients were in euthyroidism status before RFA. The clinical and imaging features are demonstrated in **Table 2**. The most common symptoms were sudden neck swelling, erythema, and pain, and one patient reported fever. The mean time from RFA to nodule rupture was 68.6 ± 55.7 days (range, 16 to 195). 3 patients were initially diagnosed with computed tomography (CT), these patients initially went to the emergency department to seek treatment. The other 6 patients were evaluated and diagnosed by US following the development of symptoms. The anterior type was the most common type of rupture (8/9, 89%), which was characterized by anterior nodule capsule disruption; CT revealed heterogeneous density of soft tissue bulging to the anterior side of the ablated nodule and tracheal deviation, which was shown in **Figure 1**.

**Table 1:** Baseline characteristics of the 9 patients with thyroid nodule rupture after RFA.

Basic Characteristics					RFA			
Case No.	Age	Sex	Size (mm)	Volume (ml)	Times	Procedure Time	Max of RF power (W)	Total energy (kCal)
1	27	F	69 x 58 x 42	88	2	28 min 40 sec, 21 min 50 sec	120, 40	33.34, 6.54
2	44	F	81 x 79 x 59	198	1	50 min 43 sec	50	25.14
3	42	F	27 x 26 x 21	7.7	1	9 min 23 sec	30	1.5
4	30	F	80 x 55 x 51	117	2	39 min 36 sec, 47 min 26 sec	60, 60	27.13, 24.43
5	46	M	54 x 39 x 36	39.6	1	22 min 50 sec	50	12.9
6	48	F	42 x 34 x 22	16.4	1	24 min 32 sec	30	8.02
7	32	F	26 x 33 x 36	16.2	2	26 min 21 sec, 22 min 07 sec	45, 70	7.41, 8.36
8	45	F	37 x 37 x 37	26.5	1	28 min 21 sec	60	16.98
9	36	M	63 x 53 x 89	155.6	2	25 min 38 sec, 30 min 07 sec	90, 60	25.16, 19.26

**Table 2:** Clinical and imaging manifestations of the 9 patients with thyroid nodule rupture after RFA.

Case No.	Time to rupture (days)	Tool of Diagnosis	Symptoms	Type	Initial management	Hospitalization/days	Intervention	Outcome
1	116	US	Sudden neck swelling with fever	Medial	Antibiotics and observation	Y/16	I&D	Complete recovery
2	19	CT	Neck erythema	Anterior	Antibiotics and observation	Y/7	Surgical debridement	Complete recovery
3	39	US	Sudden neck swelling with erythema	Anterior	Antibiotics and observation	Y/2	I&D	Complete recovery
4	195	CT	Progressive neck erythema and swelling	Anterior	Antibiotics and observation	Y/20	Surgical debridement	Complete recovery
5	71	US	Sudden neck erythem and swelling	Anterior	Antibiotics and observation	N	I&D	Complete recovery
6	97	US	Sudden neck swelling	Anterior	Antibiotics and observation	N	N	Complete recovery
7	20	US	Sudden neck swelling and pain	Anterior	Antibiotics and observation	N	N	Complete recovery
8	20	CT	Sudden neck swelling and pain	Anterior	Observation only	N	N	Complete recovery
9	40	US	Sudden neck swelling and pain	Anterior	Observation only	N	Aspiration	Complete recovery

US, ultrasound; CT, computed tomography; I&D, incision and drainage.

Only 2 patients were initially treated by analgesics, followed by close observation. Meanwhile, 7 patients (78%) received antibiotics treatment initially, and 6 of them required subsequent invasive procedures, including aspiration (1), incision and drainage (3), and surgical debridement (2). 4 patients required hospitalization, with the duration between 7-20 days. None of the patients experienced hypothyroidism after RFA. The review of literatures was presented in **Table 3**. After combining the data of 9 patients in the 2 of our institutions with data of other 17 patients extracted from the previously published studies (10, 12), the data of 26 patients (mean age 41.5 ± 14.3 years, 17 females, 9 males) were evaluated. There were 13 patients receiving conservative therapies, and the other 13 ones required invasive management. The average duration to rupture was 54.8 ± 43 days. Most patients with rupture were diagnosed *via* ultrasound (23/26, 88.5%), which reflects its convenience and utility as a follow-up tool. Symptoms were primarily neck swelling and pain, while 4 patients suffered neck erythema and 2 presented with fever (7.7%). The anterior rupture was the most common type (22/26, 84.6%).

**Table 3:** Literature review of goiter rupture after RFA for benign nodules.

First Author, Year (Ref.)	Country	Total RFA sessions	Complication rate of rupture (Percentage/patients)	The largest Diameter of ruptured nodule (mm)	Mean Volume of ruptured nodules (ml)	Mean Time to rupture (Day)	Percentage of anterior rupture	Invasive management
Present study		791	1.1% (9)	57.2 ± 21.7	73.9 ± 65.5	68.6 ± 55.7	89% (8/9)	67% (6/9)
Shin 2011 (10)	Korea	2616	0.2% (6)	41.8 ± 13.5	12.3 ± 18.3	38.5 ± 19.5	100% (6/6)	50% (3/6)
Baek 2012 (6)	Korea	1543	0.19% (3)	–	–	33.3 ± 12.5	100% (3/3)	33% (1/3)
Valcavi 2015 (19)	Italy	40	2.5% (1)	–	–	26	–	Nil (0/1)
Che 2015 (20)	China	375^a^	0.27% (1)	–	–	7	–	Nil (0/1)
Kim 2016 (11)	Korea	746	0.4% (3)	–	37.5 ± 24.2	3 months^b^	–	33% (1/3)
Chung 2019 (12)	Korea	–	(12)^c^	37.3 ± 15.7	17.2 ± 20.1	54.6	67% (8/12)	33% (4/12)

The complication of nodule rupture is 0.2-2.5%. Invasive management included aspiration, I&D, and surgical debridement/lobectomy; ^a^The study enrolled patient with benign nodule and recurrent thyroid malignancy simultaneously. Only patient with benign thyroid nodules were discussed here; ^b^The accurate days was not mentioned in the study; ^c^The total number of patients receiving RFA was not mentioned in the article.

Nonparametric statistics identified significant differences in nodule diameter and the ablation time of the course prior to rupture (RUP time) between the two groups (p = 0.045 and 0.008, respectively) (**Table 4**). The p-value of the nodule volume was marginally significant (p = 0.054), which may be limited by the relatively small sample size and maintained after further regression analysis. Two-tailed Pearson’s correlation revealed the initial nodule volume is moderately correlated with RUP time (r = 0.643), with high statistical significance (p <0.001). Logistic regression analysis indicated the nodule diameter and RUP time are associated with the requirement of invasive management, with OR 1.99 (1.07-3.67, p = 0.029) and 1.11 (1.01-1.22, p = 0.025) respectively (additional data are given in online resource, **Supplementary Table 1**). Based on the ROC curve analysis, when a nodule with the initial maximum diameter of >4.5cm ruptured, invasive management was suggested, with sensitivity of 69% and specificity of 79% (the area under curve, AUC 0.75). If the ruptured nodule occurred with an ablation time of >20mins, invasive management was suggested, with sensitivity of 62% and specificity of 69% (AUC 0.81). However, an ablation time of >20 mins appears not to be uncommon for a single RFA session. This has led to the diminished clinical application significance of this parameter. Therefore, we will focus on the impact of initial nodule size in the following discussion. All patients recovered without long-term sequelae.

**Table 4:** Characteristics that determine if aggressive management is needed (results after collating other literatures^a^).

	Need invasive management	Need for conservative management only	p Value
Number	13	13	
Age (year)	41.0 (28.5-50.0)	42.0 (39.0-40.0)	0.681
Gender (female)	54% (7/13)	77% (10/13)	0.216
Diameter (cm)	5.4 (3.3-7.5)	3.7 (2.9-4.5)	0.045*
Volume (mL)	39.7 (7.3-102.7)	16.2 (5.9-22.5)	0.054
Solid content of nodule^b^	85% (11/13)	77% (10/13)	0.315
RUP time(min:sec)	24:50 (14:07-40:00)	11:13 (6:00-21:34)	0.008*
Max RF power of the session of nodule rupture	60.0 (50.0-70.0)	55.0 (35.0-77.5)	0.677
Time to rupture (day)	40 (22-76)	48 (20-63)	0.797

The diameter of nodule and ablation times both revealed significant difference between the two groups that need aggressive managements or note. Data were expressed as median (0.25-0.75 quartile). The P value was calculated from Mann Whitnet U test and < 0.05 was considered significant; RUP time: the ablation time of the course prior to rupture; ^a^Patients data obtained from two articles (10, 12). ^b^One patient from the study of Shin et al. (10) was with record of spongiform nodule. *p value with statistical significance (< 0.05).

## Discussion

Despite the frequently favorable prognosis after nodule rupture, some patients still require hospitalization for administration of intravenous antibiotics, or possible surgical intervention. If these patients can be identified earlier, it will result in reduced medical expenses and increased patient satisfaction. The present retrospective study enrolled patients from the 2 major thyroid RFA medical centers in the country. Furthermore, the literature review that identified patients having suffered nodule rupture post RFA and factors related to the determination of treatment methods were investigated. Notably, this study revealed that a larger initial nodule size and longer procedural time were associated with increased need for invasive management.

Previous studies have reported the complication rate of ablated nodule rupture was 0.19-2.5% (**Table 3**). In one study which enrolled 1459 patients treated with RFA, the complication rate of nodule rupture was 0.19% (3/1543 nodules); indeed, the size of the ablated nodule had gradually decreased prior to rupture (6). The diagnosis of nodule rupture is based on clinical symptoms and images; while the most common symptom is sudden painful bulging neck mass (10, 12). In our study, the common symptoms were sudden neck swelling (89%), erythema (50%), and pain (33%). The US features included breakdown of the thyroid capsule with formation of fluid accumulation or new mass, indicating the interaction between intra- and extra-thyroidal spaces. Hematoma or intra-nodular hemorrhage during RFA can be controlled by direct compression and/or direct ablation at the bleeding point with the RF electrode (18). In 2019, various types of rupture were classified according to localization of rupture by Chung et al. (12). Although the anterior type was the most common, it was typically exhibited in the strap muscle layer. By comparison, with the contents bulging medially toward to the tracheoesophageal (TE) groove, the medial type may cause a mass effect on the trachea, leading to a compromised airway.

As compared to a multicenter retrospective study in Korea which included 1491 patients with 2616 RFA sessions wherein six cases (0.2%) with rupture were reported (10), an acceptable rupture rate was noted in our study. By comparing our patients with the 17 patients identified from previous studies (10, 12), a younger patient age (38.9 ± 7.8 *vs*. 39.4 ± 15.3, p 0.033) and larger ruptured nodule size (diameter 5.7 ± 2.3 *vs*. 4.1 ± 1.4 cm, p 0.018 and volume 73.9 ± 69.4 *vs*. 20 ± 20.8 ml, p <0.001) were noted in patients of the present study, which may be related to patient selection (**Table 5**). Additionally, the RUP time was longer (29.3 ± 13.5 *vs*. 16.2 ± 11.8 mins, p= 0.896), and the median energy per RFA session was lower (50 (35-60) *vs*. 60 (50-85) W, p =0.16 with nonparametric statistics). The above findings reflect the current status of nodule rupture management, providing a valuable and practical reference for clinicians. Since larger nodule size and longer procedure time are associated with an increased need for invasive management upon rupture event, when a patient’s condition meets the abovementioned clinical criteria, initial intervention is recommended.

**Table 5:** Comparison of patient characteristics of our study and other previous studies.

Source of patients data	Present study	Previous studies	p Value
Number	9	17	
Age (year)	38.9 ± 7.8	39.4 ± 15.3	0.033*
Gender (female)	7(78%)	10(59%)	0.216
Diameter (cm)	5.7 ± 2.3	4.1 ± 1.4	0.018*
Volume (mL)	73.9 ± 69.4	20 ± 20.8	<0.001*
Solid content of nodule	9(100%)	12(71%)^a^	0.07
RUP time (min:sec)	29.3 ± 13.5	16.2 ± 11.8	0.896
Max RF power of the session of nodule rupture	50.0(35.0-60.0)	60.0(50.0-85.0)	0.16
Time to rupture (day)	69 ± 59	48 ± 35	0.068

By comparing the 9 patients with the 17 patients identified from previous studies (10, 12), a younger patient age (38.9 ± 7.8 vs. 39.4 ± 15.3, p 0.033) and larger ruptured nodule size (diameter 5.7 ± 2.3 vs. 4.1 ± 1.4 cm, p 0.018 and volume 73.9 ± 69.4 vs. 20 ± 20.8 ml, p <0.001) were noted in the presented patients, which may be related to patient selection. Data were expressed as mean ± standard deviation except for the Max RF power, which with non-normal distribution. It was expressed as median (0.25-0.75 quantile); The P value was calculated from independent t-test and < 0.05 was considered significant, except for the parameter of RF power, which was calculated from Mann Whitnet U test. RUP time: the duration of the RFA session of nodule rupture; ^a^One patient from the study of Shin et al. was with record of spongiform nodule. *p value with statistical significance (< 0.05).

We compared the characteristics of our 9 patients, basing on need and not need of invasive management (6 and 3 patients, respectively), and no significant difference was noted on nodule diameter (p= 0.905), ablation time (p= 0.905), RF power (p= 0.548) nor time to rupture (p= 0.548). A longer duration between the RFA and rupture event was noted in the patients from our institutions (68.6 ± 55.7 *vs*. 47.5 ± 34.6 days, p 0.068), indicating that for larger nodules, well-timed and longer duration of follow-ups are necessary. Furthermore, detailed postoperative explanations, including self-care and vigilance of risk signs, including fever, sudden pain, and erythema should be communicated to the patient.

RFA leads to the formation of a fibrous capsule around the area of the coagulative necrosis, while central scattered hyaline sclerosis and scarring can be observed microscopically (21, 22). In addition, spontaneous intra-nodular hemorrhage may occur under pressure (such as coughing, crying, neck pressure or twisting) (23). The possible mechanisms include abnormal vessel anatomy with weakening of the veins and intra-nodular arterio-venous shunting, resulting in extravasation into the nodule under pressure, especially for patients with hypervascularity prior to RFA (24, 25). However, the cause of nodule rupture after RFA remains unclear, and is not necessarily related to trauma or delayed bleeding. Furthermore, the use of higher energy during the procedure, larger amounts of necrotic tissue after RFA, and more severe tissue edema may all contribute to higher risk of rupture for large nodules. The more obvious compression symptoms caused by a large nodule may also impel the physician to actively manage the event. One multicenter study published by Baek et al. suggests that nodule rupture may be caused by acute volume expansion due to hemorrhage, and conservative management was suggested (6). In our study, one female with a large thyroid mas (8.1 x 7. 9 x 5.9 cm) was treated with RFA. Sudden swelling and erythema was noted 19 days later, with CT demonstrating anterior nodule rupture (**Figure 1**). During debridement, large volumes of mucous and turbid content were noted, without delayed hemorrhage. Therefore, imaging can be an effective diagnostic tool, however the evaluation of delayed bleeding primarily depends on the surgical or aspiration findings. Of the 9 patients in our study, 3 were prescribed oral analgesics, and 1 with oral steroid. 7 patients initially received treatment with antibiotics, but the improvements were limited; 4 patients required hospitalization for intravenous antibiotics administration, and 6 patients required subsequent invasive procedures.

This is currently the most comprehensive study to analyze the factors determining invasive treatment for nodule rupture after RFA. Still our findings should be interpreted in light of some limitations. Although the nodular size will affect the treatment, whether the use of steroids or other medications to reduce tissue swelling can reduce the hospitalization days or even prevent rupture requires further verification. The low probability of nodule rupture after RFA and diverse management modalities may mask unknown factors that can affect further treatment. Certainly, these two factors identified (initial nodule volume and ablation time) would probably not be significant, if applied in our own cohort of 818 patients or future larger cohort. Larger scale, multicenter studies will be necessary for more detailed evaluation. However, even a limited number of patients can yield meaningful results; we believe the results herein can be generally applied, at least for the Asia patients. In addition, the clarification of correlations between rupture, preclinical history, and laboratory data changes (white blood cell count, high sensitivity C-reactive protein, or erythrocyte sedimentation rate) should be a focus of future research.

## Conclusion

The determination of treatment of post-RFA ruptured nodule depends on the nodule size and procedure time. Though some ruptured nodules can be managed conservatively, those with a maximum initial diameter of >4.5cm may necessitate more invasive management. Understanding these features with appropriate risk assessment will help physicians to accurately diagnose and to improve patient’s satisfaction.

## Data Availability Statement

The raw data supporting the conclusions of this article will be made available by the authors, without undue reservation.

## Ethics Statement

The studies involving human participants were reviewed and approved by the institutional review boards of Kaohsiung Chang Gung Memorial Hospital and the institutional review boards of Chung Shan Medical University Hospital. Written informed consent for participation was not required for this study in accordance with the national legislation and the institutional requirements.

## Author Contributions

For the contribution of each author, Wen-CC and W-CL conceived and designed the analysis. Wen-CC and K-LC collected the data. Y-HC and C-KC contributed data or analysis tools. Wei-CC and S-DL performed the analysis. Wen-CC and K-LC wrote the paper. All authors contributed to the article and approved the submitted version.

## Conflict of Interest

The authors declare that the research was conducted in the absence of any commercial or financial relationships that could be construed as a potential conflict of interest.

## Publisher’s Note

All claims expressed in this article are solely those of the authors and do not necessarily represent those of their affiliated organizations, or those of the publisher, the editors and the reviewers. Any product that may be evaluated in this article, or claim that may be made by its manufacturer, is not guaranteed or endorsed by the publisher.
