# Supplementary material for: The Importance of Nodule Size in the Management of Ruptured Thyroid Nodule After Radiofrequency Ablation: A Retrospective Study and Literature Review
Source: Front Endocrinol (Lausanne). 2021 Nov 26;12:776919. doi: 10.3389/fendo.2021.776919 (PMC8662308; doi:10.3389/fendo.2021.776919)
Supplement: Supplementary file 1 [file Table_1.docx]

**Supplementary table 1.** Odds ratios of the requirement of invasive management associated with nodule rupture

|  | Odds ratio (95%CI) | P value |
| --- | --- | --- |
| Diameter (cm) | 1.99(1.07-3.67) | 0.029 |
| Volume (mL) | 1.05(0.998-1.10) | 0.063 |
| AVE time | 1.11(1.02-1.22) | 0.022 |
| RUP time | 1.11(table) | 0.025 |

Logistic regression analysis indicated that the diameter and RUP time significantly affected the requirement of invasive treatment (OR 1.99 and 1.11, respectively).
